# Too tired to learn: insomnia, sleep quality, and sleep aid practices among Kasr Al Ainy medical students: a cross-sectional analysis

**DOI:** 10.1186/s12909-025-08054-1

**Published:** 2025-11-06

**Authors:** Omnia Azmy Nabeh, Rehab Magdy, Mostafa Ahmed Khalifa, Aya Magdy Siam, Aya Abdulkarim Elbhairy, Tasneem Abdelwahab, Albraa Ashraf Hamad, Mostafa Atef Diab, Soha Aly Elmorsy

**Affiliations:** Kasr Alainy Faculty of Medicine, Cairo University, Cairo, Egypt

**Keywords:** Insomnia, Medical students, Medications, Melatonin, Quetiapine, PSQI, Sleep aid

## Abstract

**Introduction:**

Medical students face a unique set of challenges, including high academic stress, irregular schedules, and clinical responsibilities, which place them at increased risk for sleep disturbance, particularly insomnia.

**Methods:**

This study aimed to assess the prevalence of insomnia using the DSM-5-TR core symptoms, evaluate sleep quality using the Pittsburgh Sleep Quality Index (PSQI), and investigate the use and safety of sleep aid medications in this population. A cross-sectional survey was conducted among 1,640 Kasr Al Ainy medical students, Cairo, Egypt. Data collected included demographic characteristics, medical history, insomnia symptoms, and PSQI scoring. Sleep aid utilization, including off-label use of medications like quetiapine, was specifically examined.

**Results:**

The mean age of participants was 20.53 ± 2.08 years, with 58.7% being female. A total of 80% reported at least one insomnia symptom, and 93.7% had poor sleep quality (PSQI ≥ 5). Psychiatric conditions were reported by 29.2% of participants, significantly more common among females (*p* < 0.001). Although 259 participants were taking medications with sedative properties, only 8.8% sought medical advice for insomnia and just 157 explicitly reported using sleep aids. Melatonin was the most used (*n* = 121), followed by herbals, eszopiclone, and diphenhydramine. Seventeen participants reported using quetiapine, of whom 13 (76.5%) experienced adverse effects. Among the 12 participants who reported on its effectiveness, 5 (41.7%) perceived it as having limited efficacy.

**Conclusion:**

This study reveals the burden of sleep disturbance among medical students, with a major gap between the prevalence of insomnia and the utilization of professional sleep interventions. The off-label use of quetiapine, even among a small subset of our sample, raises notable safety concerns. This finding serves as an important pharmacovigilance signal and underscores the need for further investigation into the risks associated with this potentially hazardous practice. Non-pharmacological strategies such as sleep hygiene education and cognitive-behavioral interventions are needed.

**Supplementary Information:**

The online version contains supplementary material available at 10.1186/s12909-025-08054-1.

## Introduction

Medical students are a unique population frequently exposed to high academic stress, irregular schedules, and demanding responsibilities. The competitive nature of medical education and the endless journey of learning and rescuing patients, together with the stressful working shifts, place a significant burden on their physical, mental, and social well-being [1].

Insomnia and poor sleep quality are other distressing concerns among medical students, being alarmingly prevalent [2–4]/According to the Diagnostic and Statistical Manual of Mental Disorders criteria (DSM-5) published in 2013, insomnia is diagnosed by symptoms of difficulty in falling asleep, staying asleep, or achieving restorative sleep [5]. Furthermore, sleep quality is commonly evaluated using the Pittsburgh Sleep Quality Index (PSQI) (Buysse et al. 1989) [6]. This index consists of 19 self-rated questions that evaluate seven aspects to judge sleep quality. These aspects are grouped as subjective sleep quality, sleep latency, sleep duration, sleep efficiency, sleep disturbances, sleep medications, and daytime dysfunction. In 2016, Puthran et al. reported in their systematic review that 52.7% of medical students suffer from poor sleep quality, attributed to stress, extended study hours, and irregular schedules [7]. These disturbances are strongly associated with diminished academic performance.

To address sleep disturbances, medical students often turn to sleep aids use, including both prescription and over-the-counter (OTC) medications, without proper medical guidance. This practice increases the risk of adverse effects and dependency, particularly with off-label use of some medications like the antipsychotic medication, quetiapine [8]. Although quetiapine is commonly used off-label to manage sleep disturbances, its efficacy, dependency potential, and adverse effects raise significant concerns [9].

On the other hand, many students resort to caffeinated drinks, tobacco, and energy drinks to stay awake and study, which can further disrupt their sleep quality and negatively impact their overall health [10].

An additional layer of complexity among medical students arises regarding the management of these conditions. The self-perception of being a healthcare provider often leads medical students to self-diagnose and self-medicate themselves without seeking proper medical consultation. This practice, derived from overconfidence or a perceived stigma around seeking help, increases the risk of inappropriate medication use and potential complications [8]. These paradigms emphasize the need for structured education and awareness protocols about medication-use safeguards, especially for sleep aid.

This cross-sectional study aimed to conduct a pharmacoepidemiologic analysis of insomnia prevalence, sleep quality, and the utilization of sleep aid medications among medical students at Kasr Al Ainy Faculty of Medicine, Cairo, Egypt.

## Experimental procedures

This study was conducted between October and December 2024 using a web-based survey targeting medical students at Kasr Al-Ainy, Cairo University, Egypt. The study protocol received approval from the Research Ethics Committee (REC) of the Faculty of Medicine, Cairo University (Approval No. N.263.20204) and was conducted in accordance with the principles of World Medical Association Declaration of Helsinki [11]. In addition to the standardized Pittsburgh Sleep Quality Index (PSQI) items, a customized questionnaire was developed specifically for this study (see Supplementary File: Study Questionnaire). The survey was distributed via WhatsApp groups and Facebook pages commonly used by the target population. Participation was voluntary, and electronic informed consent was obtained from all participants prior to survey completion.

The questionnaire was designed to collect comprehensive data across multiple domains to assess: demographic characteristics; prevalence of relevant disorders (particularly neuropsychiatric disorders and chronic illnesses; medication use among participants; insomnia symptoms assessed according to the DSM-5 Core Diagnostic symptoms [5], insomnia management strategies and the use of sleep aid, defined as any medication self-reported by participants to be used specifically for managing sleep difficulties, regardless of their primary indication; quetiapine use and perceived efficacy; beverage and tobacco consumption habits; and finally, the Pittsburgh Sleep Quality Index (PSQI) analysis [6].

The PSQI consists of 19 items grouped into seven components, each representing a specific aspect of sleep. Each component is scored on a 0 to 3 scale, where 0 indicates no difficulty and 3 indicates severe difficulty. The global PSQI score is the sum of all component scores, ranging from 0 to 21. A global score greater than 5 suggests clinically significant sleep disturbances, with higher scores indicating poorer sleep quality. Each component of the PSQI represents the score for one or more of its 19 items. The components include: *subjective sleep quality*, which is rated based on the overall perception of sleep quality; *sleep latency*, assessed as the sum of scores for the time it takes to fall asleep and the frequency of difficulty initiating sleep, and scored as 0 points for a combined score of 0, 1 point for a combined score of 1–2, 2 points for a combined score of 3–4, and 3 points for a combined score of 5–6. *Sleep duration* is determined by the total hours of actual sleep and scored as 0 points for ≥ 7 h, 1 point for 6–7 h, 2 points for 5–6 h, and 3 points for < 5 h. *Habitual sleep efficiency* is calculated as the percentage of time spent asleep relative to time in bed and scored as 0 points for ≥ 85%, 1 point for 75%–84%, 2 points for 65%–74%, and 3 points for < 65%. *Sleep disturbances* are scored based on the sum of the frequency of interruptions, such as waking during the night, needing the bathroom, breathing difficulties, or discomfort, and are scored as 0 points for a combined score of 0, 1 point for a combined score of 1–9, 2 points for a combined score of 10–18, and 3 points for a combined score of 19–27. The *use of sleep medications* is scored based on the frequency of taking medications to aid sleep. Lastly, *daytime dysfunction* is evaluated through the sum of scores from two questions assessing difficulties staying awake during activities and maintaining enthusiasm, with scores converted into a single component as follows: 0 points for a combined score of 0, 1 point for a combined score of 1–2, 2 points for a combined score of 3–4, and 3 points for a combined score of 5–6.

Data analysis was conducted using SPSS version 26 and R software [12], utilizing libraries such as *dplyr* for data manipulation and *stats* for statistical analysis. Categorical data were presented as frequencies and percentages. Numerical data were reported as mean ± standard deviation (SD) for normally distributed variables and as median (range) for non-normally distributed variables. Comparisons of numerical data were performed using t-tests, and categorical data were analyzed using the Chi-square (χ^2^) test. A p-value of less than 0.05 was considered statistically significant [13–15]. Data visualization was carried out using Microsoft Excel, Python, and R software. In Python, visualizations were created using libraries such as Matplotlib and Seaborn, while in R, the *ggplot2* library was employed (R Core Team**).** A subgroup analysis was planned for participants reporting quetiapine use, including descriptive statistics of dose, adverse effects, and concurrent chronic illnesses. Pharmacovigilance outcomes among quetiapine users were, also evaluated using a confounder-adjusted descriptive approach, including stratification and Fisher’s Exact Test, due to the limited sample size. Additionally, potential confounding factors—such as concurrent use of other sedative medications and the presence of psychiatric disorders—were qualitatively examined.

## Results

### Participants demographics

**Fig. 1:**
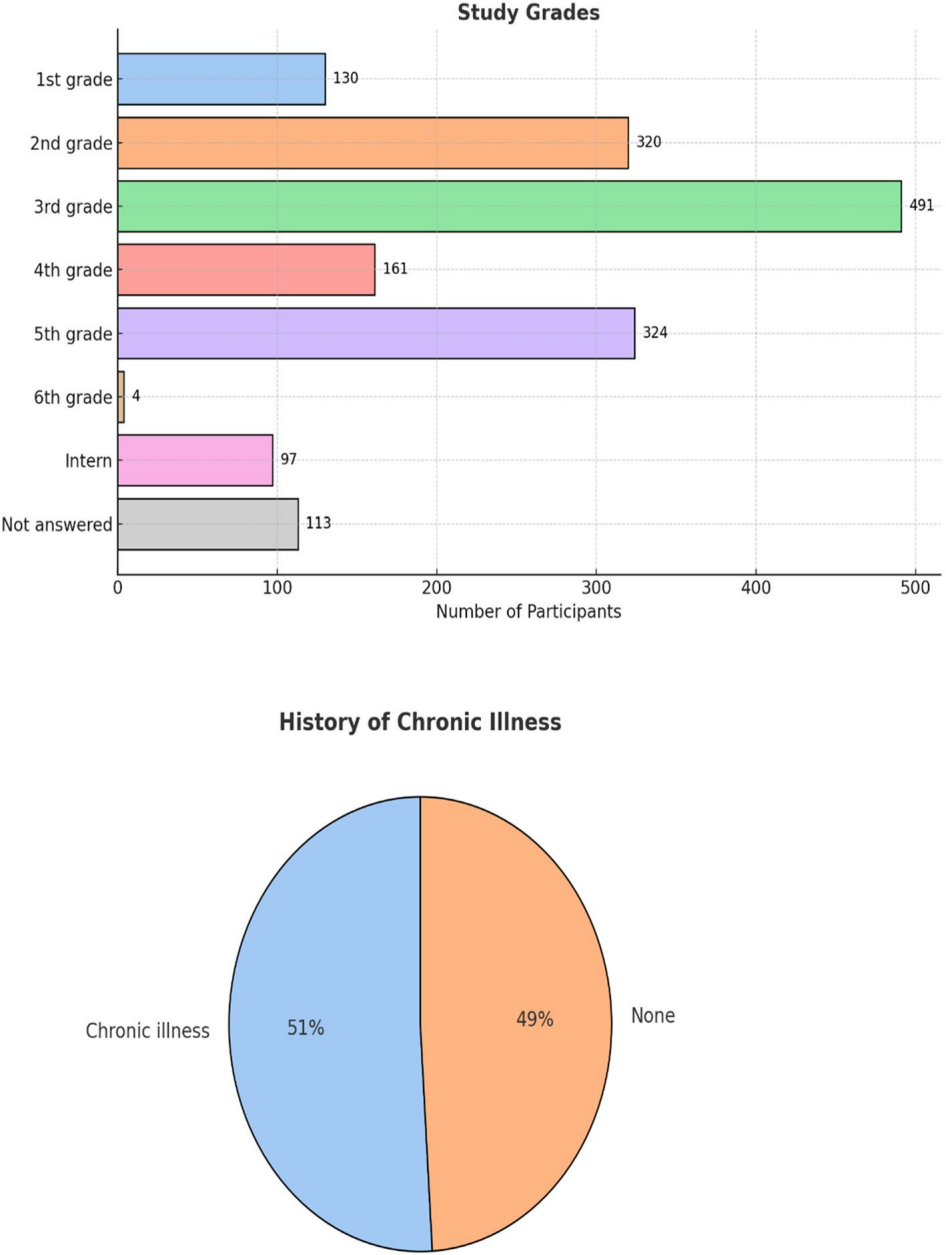
Upper Panel (Bar Chart): Frequency distribution of participants by study grade. Lower Panel (Pie Chart): Proportion of participants with and

The study included a total of 1,640 participants, out of which 963 participants (58.7%) were female, while 677 participants (41.3%) were male. The participants' ages ranged from 16 to 30 years, with a mean age of 20.53 years and a SD of 2.08 years. Students from different studying grades participated in the questionnaire, as shown in Fig. 1.

### Prevalence of chronic illnesses

As regards the history of chronic illness, the results showed that 51% of the participants reported having at least one chronic illness and 473 participants (28.8%) reported having more than one chronic illness. The most frequently reported chronic illness was anxiety (410 cases, 25.0%), followed by anemia (244 cases, 14.9%) and depression (183 cases, 11.2%).

Furthermore, a total of 480 participants (29.2%) reported having at least one of the following psychiatric illnesses: anxiety, depression, obsessive compulsive disorder (OCD), attention-deficit hyperactivity disorder (ADHD), obstructive sleep apnea, or other psychiatric conditions. Of this group, 340 (70.8%) were female [16], and 139 (28.9%) were male, indicating a significant sex disparity in the prevalence of psychiatric disorders among the participants (*p* < 0.001).

### Medication use among participants

When asked if they were receiving any medications for their medical conditions, 259 participants stated taking regular medications to control their chronic illness (Table 1). The most commonly used medication class was the “*Iron, vitamins, and supplements”*. *Anti-depressant* class was also widely used (*n* = 59), ranked the second, with sertraline, escitalopram, and fluoxetine being the most frequently reported medications. The use of other medications with sedative adverse effects such as: antihistaminics (*n* = 22), anti-epileptics (*n* = 6), antipsychotics (*n* = 5), sedative-hypnotics (*n* = 4), mood stabilizers (*n* = 2) were also reported.

**Table 1:** Frequency of medication class usage among participants

Medication Class	Usage frequency
Iron, vitamins, and supplements	76
Anti-depressant	59
Asthma medications	30
For gastroesophageal reflux disease (GERD)	23
Cardiovascular	22
Allergy/Sinusitis and Anti-histaminic	22
Thyroid	20
Anti-diabetics	11
For irritable bowel syndrome (IBS)	9
Immunosuppressives and disease modifying anti-rheumatic drugs (DMARDs)	8
Eye medications/optics	7
Anti-epileptic	6
Painkillers	6
Anti-psychotic	5
Sedative-hypnotic (Sleep aid)	3
Blood disorders	2
Mood stabilizer	2
Sedative-hypnotic (Sleep aid) and for restless leg syndrome	1
Others	1

### Insomnia symptoms and management

As regards insomnia symptoms, a total of 1299 participants (79.2%) reported at least one symptom of insomnia, including [e.g., difficulty initiating or maintaining sleep, early awakening], as assessed by a customized insomnia symptom checklist based on DSM-5 criteria. Among them, 1067 individuals (65%) experienced more than one insomnia symptom. The most frequently reported symptoms were: “still feeling tired after waking up” with 941 participants, “feeling tired and irritable during the day” with 704 participants, and “difficulty concentrating” with 655 participants. Other commonly reported symptoms included “difficulty falling asleep” with 602 participants and “lying awake at night” with 516 participants. Less frequently reported symptoms included “waking up too early” with 212 participants, “having ongoing worries about sleep” with 361 participants, and “finding it hard to nap during the day despite being tired” with 371 participants.

### Pittsburgh Sleep Quality Index (PSQI) analysis

After thorough data cleaning and the removal of missing entries, valid Pittsburgh Sleep Quality Index (PSQI) responses were available for analysis from 619 participants, comprising 319 females and 300 males. The results of the PSQI analysis are summarized in Table 2. The mean global PSQI score was 9.63 ± 2.8, and with scores ranging from 0 to 19. Notably, 93.7% of participants were classified as having poor sleep quality (PSQI ≥ 5).

**Table 2:** Summary of Pittsburgh Sleep Quality Index (PSQI) metrics among participants

Metric	Mean	SD	Min–Max
Global PSQI Score	9.63	2.8	0–19
Global PSQI Score (males n = 300)	9.73	2.71	0–19
Global PSQI Score (females n = 320)	9.51	2.91	0–17
Subjective Sleep Quality (0–3)	2.6	0.85	0–3
Sleep Latency (minutes)	45.76	38.47	5–180
Sleep Latency (0–3)	1.57	0.88	0–3
Sleep Duration (hours)	6.43	1.72	4–10
Sleep Duration (0–3)	1.4	1.15	0–3
Habitual Sleep Efficiency (0–3)	1.38	1.17	0–3
Sleep Disturbances (0–3)	1.28	0.62	0–3
Use of Sleep Aid Medications (0–3)	0.2	0.58	0–3
Daytime Dysfunction (0–3)	1.34	0.97	0–3

For the individual components, the mean score for subjective sleep quality was 2.6 ± 0.85. Sleep latency had a mean duration of 45.76 min (range: 5–180 min), corresponding to a mean component score of 1.57 ± 0.88. The mean sleep duration was 6.43 ± 1.72 h, with a mean component score of 1.4 ± 1.15. Habitual sleep efficiency was reported with a mean component score of 1.38 ± 1.17, while sleep disturbances had a mean score of 1.28 ± 0.62. Use of sleep aid medications was infrequent, reflected by a mean score of 0.2 ± 0.58. Daytime dysfunction had a mean score of 1.34 ± 0.97.

**Fig. 2:**
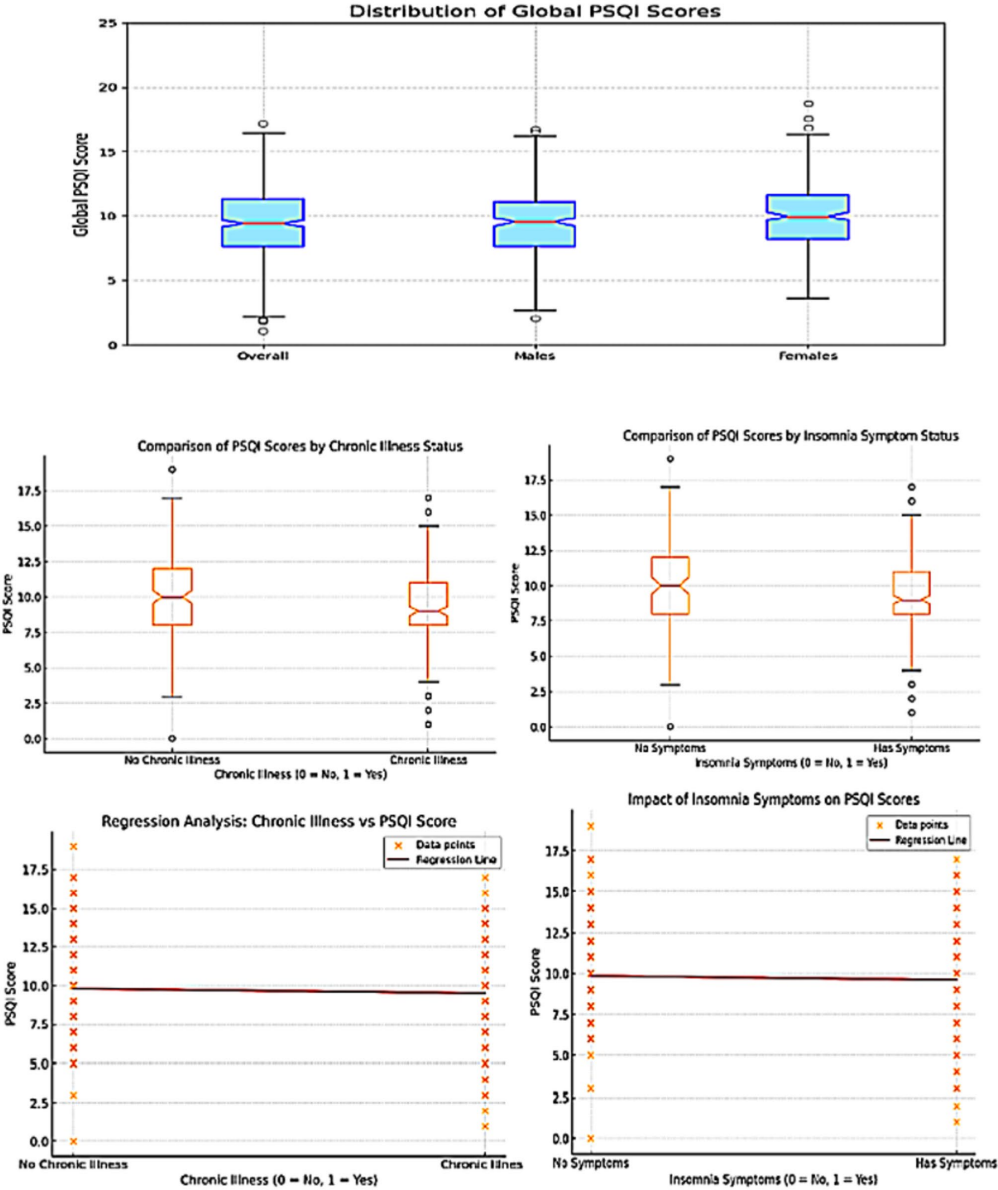
Analysis of Global PSQI Scores by Chronic Illness and Insomnia Symptoms. Top Panel: Distribution of Global PSQI Scores overall, and stratified by sex (males and females). Box plots illustrate the median, interquartile range, and outliers of the scores across these groups. Middle Left: Comparison of PSQI scores between individuals with and without chronic illness. Middle Right: Comparison of PSQI scores between individuals with and without insomnia symptoms. In both figures, both groups showed a similar spread in PSQI scores, with overlapping ranges, reflecting the lack of a significant difference. Bottom Left: Regression analysis examining the relationship between chronic illness and PSQI scores. The regression line intercept indicates a baseline PSQI score of 9.5861 when no chronic illness is present. The coefficient for chronic illness is 0.1003, indicating a negligible increase in PSQI score associated with the presence of chronic illness. The *p*-value for the effect of chronic illness on PSQI scores is 0.657, showing no statistically significant relationship. Bottom Right: Regression analysis evaluating the impact of insomnia symptoms on PSQI scores. The regression line intercept indicates a baseline PSQI score of 9.7245 when insomnia symptoms are absent. The coefficient for insomnia symptoms is −0.1608, suggesting a minimal decrease in PSQI score associated with the presence of insomnia symptoms. The p-value for the effect of insomnia symptoms on PSQI scores is 0.48, showing no statistically significant relationship. In both figures, the regression lines in both figures appear nearly flat, indicating little to no change in PSQI scores between the two groups

When comparing global PSQI scores between males and females, no statistically significant difference was observed. The mean global PSQI score for males was 9.73 ± 2.71, while for females it was 9.51 ± 2.91, p = 0.974. Additionally, linear regression analysis revealed no detectable correlation between the presence of either chronic illness or insomnia symptoms and the PSQI score. For chronic illness, the coefficient was 0.1003 (*p* = 0.657), indicating a negligible increase in PSQI score. For insomnia symptoms, the coefficient was **−**0.1608 (*p* = 0.48), suggesting a minimal decrease in PSQI score. Both results were not statistically significant (Fig. 2).

### Beverage and tobacco consumption habits

Participants were asked about their beverage and tobacco consumption habits. Among the 1796 answers obtained for this question, the most frequently consumed items were coffee in its various forms (1037 participants), followed by black/green tea (893 participants). Five hundred eighty-four and 505 participants reported consuming sodas and cocoa/chocolate drinks, respectively. Notably, 365 participants stated that they consumed energy drinks, 91 stated being tobacco smokers, and 19 participants reported drinking alcohol. A significant portion of participants selected more than one beverage (*n* = 1312), while 407 individuals stated that they did not consume any of the listed items.

### Insomnia management and sleep aids

**Fig. 3:**
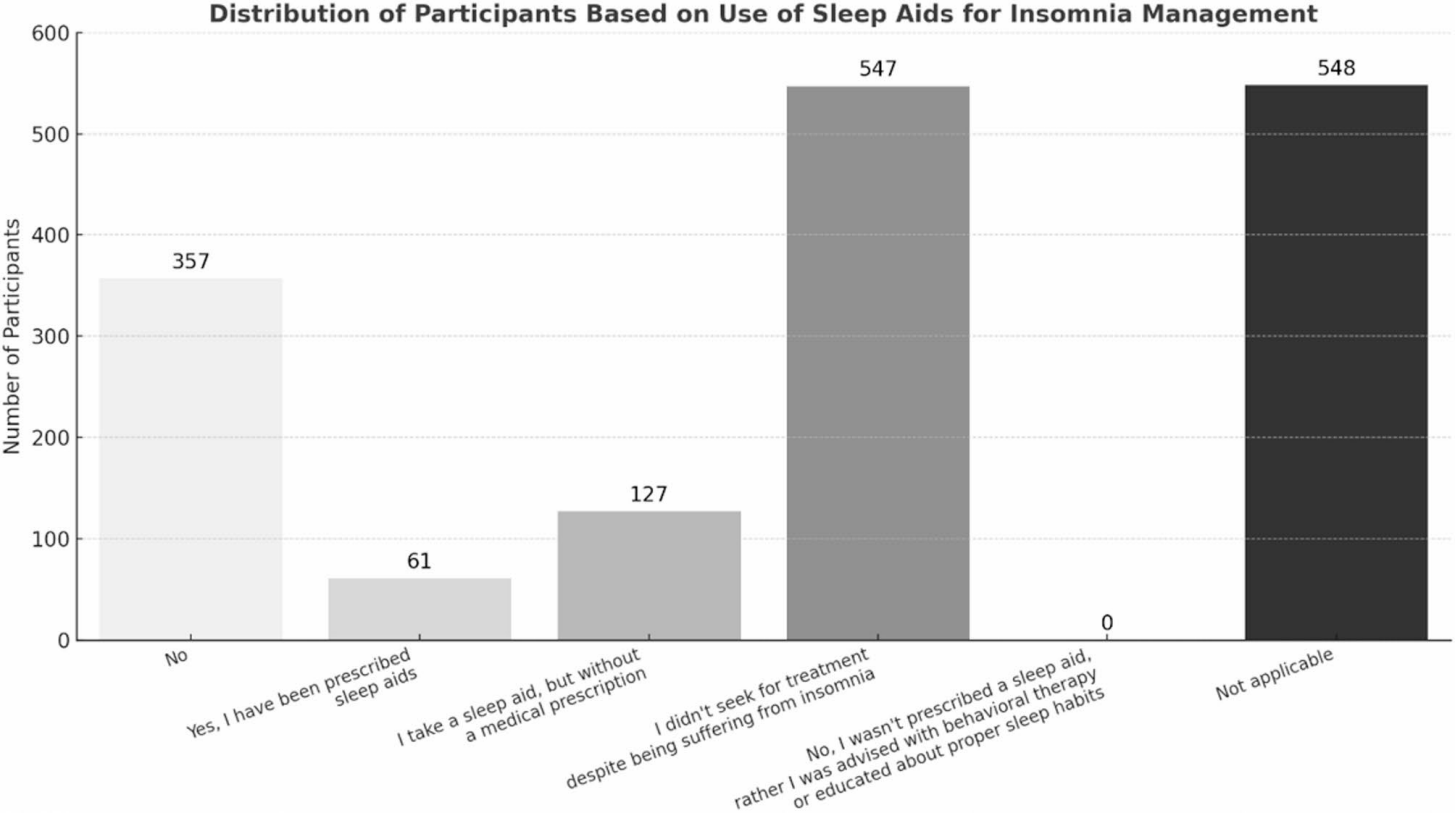
Distribution of participants based on use of sleep aids for insomnia management

Among the 1299 participants who reported experiencing insomnia, 114 individuals, representing 8.8%, sought medical advice for their conditions, while the majority, 1189 participants (91.2%), did not. The distribution of participants based on the use of sleep aids for insomnia management and the frequency of sleep aid medications use are detailed in Fig. 3.

**Fig. 4:**
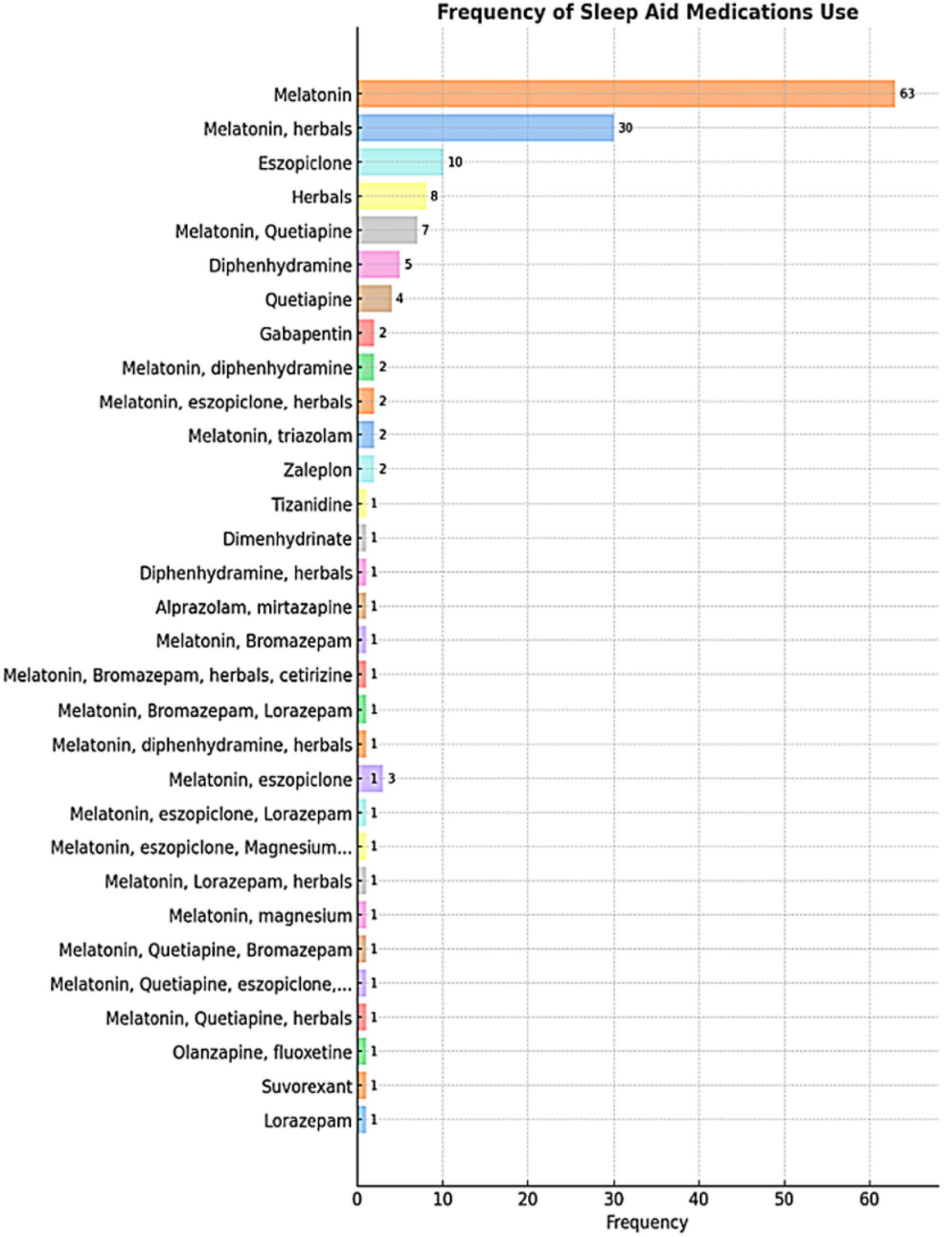
Bar charts illustrating the usage patterns of sleep aid medications

A total of 157 participants provided details about their sleep aid medications, as shown in Fig. 4. Data revealed that melatonin was the most frequently used medication (listed 121 times), followed by herbals (*n* = 46), eszopiclone (*n* = 19), and quetiapine (*n* = 14). Other listed sleep aid medications were diphenhydramine, gabapentin, triazolam, zaleplon, dimenhydrinate, suvorexant, lorazepam, and various combinations. The majority of participants (*n* = 103) reported using sleep aid medications "on demand".

### Quetiapine use and effects

As detailed in Table 3, when participants were asked if they had tried quetiapine before as a sleep aid, 14 participants answered with “yes”, while 3 participants stated that they received/were receiving quetiapine but for other indications rather than to manage insomnia. Doses ranged from 25 to 100 mg at bedtime, with the majority (8 participants, 57%) reporting 25–50 mg doses.

**Table 3:** Medical history and sleep aid experiences of participants with prior quetiapine use

Participants	Sex	Chronic Illness	Chronic Medications list	History of Sleep Aid Medications Intake	History of Quetiapine Intake as a sleep aid	Quetiapine dose	Quetiapine-reported Adverse Effect	Self-reported Quetiapine efficacy “Did quetiapine effectively managed your insomnia?”
1	Male	None	Not available	Melatonin, Quetiapine, herbals	I received quetiapine, but for other indication	Not specified	None	Not effective
2	Female	Anemia, Anxiety	Propranolol, iron supplements, quetiapine	Melatonin, Quetiapine, Bromazepam	Yes	50 mg at bed time	Drowsiness, Lack of concentration, metabolic changes, Weight gain, Menstrual changes, Palpitations	Yes, I agree
3	Male	Asthma, Anxiety, Depression	Not available	Melatonin, Quetiapine eszopiclone, diphenhydramine, herbals	Yes	25 mg at bed time	Failure to control insomnia, Drowsiness, Sedation, Lack of concentration, Increased appetite, Weight gain	Not effective
4	Female	Thyroid disorders	Not available	Not currently receiving	Yes	25 mg at bed time	None	Yes, I strongly agree
5	Male	Anxiety, Depression, Other psychiatric illness	Not available	Melatonin, Quetiapine	Yes	100 mg at bed time	None	Not effective
6	Female	Anemia, Anxiety	Not available	Melatonin, Quetiapine	Yes	25 mg at bed time	Drowsiness, Sedation, Lack of concentration, Increased appetite, Irritable the following day	Yes, I agree
7	Female	Sinusitis, Anxiety, Depression	Not available	Melatonin, Quetiapine	Yes	25 mg at bed time	Palpitations	Yes, I agree
8	Male	Anxiety, Depression	Desvenlafaxine	Melatonin, Quetiapine	Yes	50 mg at bed time	Drowsiness, Weight gain	Not effective
9	Female	Depression, Other psychiatric illness, Restless leg syndrome	Not available	Quetiapine	Yes	50 mg at bed time	Failure to control insomnia, Drowsiness, Sedation, Lack of concentration, metabolic changes, Increased appetite, Weight gain, Extrapyramidal side effects	I’m not Sure
10	Male	None	Not available	Quetiapine	Yes	25 mg at bed time	None	Yes, I strongly agree
11	Male	Anxiety, Depression, Other psychiatric illness	Not available	Not currently receiving	Yes	100 mg at bed time	Drowsiness, Lack of concentration, metabolic changes, Increased appetite, Weight gain	Yes, I agree
12	Female	Anemia	Not available	Not currently receiving	Yes	Not specified	Failure to control insomnia	Yes, I agree
13	Female	Hypertension, Eye disorders	Not available	Not currently receiving	I received quetiapine, but for other indication	Not specified	Menstrual changes	Not answered
14	Male	None	Not available	zaleplon	I received quetiapine, but for other indication	50 mg at bed time	Failure to control insomnia, Sedation	Not answered
15	Male	Asthma	Not available	Suvorexant, Quetiapine	Yes	50 mg at bed time	Sedation	Not answered
16	Male	None	Not available	Melatonin, Quetiapine	Yes	100 mg at bed time	Increased appetite	Not answered
17	Male	Anxiety, Depression, Other psychiatric illness, Restless leg syndrome, Gastroesophageal reflux disease	Modafinil, Paroxetine	Not currently receiving	Yes	25 mg at bed time	Sedation, Lack of concentration	Not answered

Quetiapine-related adverse effects were reported by 12 participants (71%). The most common side effects included drowsiness (8 participants, 47%), weight gain (6 participants, 35%), and lack of concentration (6 participants, 35%). Regarding the effectiveness of quetiapine as a sleep aid, 5 participants (29%) reported it to be effective, 6 participants (35%) reported it as ineffective, and 1 participant (6%) was unsure. Responses were missing for 5 participants (29%).

Due to the small number of quetiapine users (*n* = 17), we conducted a confounder-adjusted analysis using descriptive stratification and Fisher’s Exact Test to explore whether sex and psychiatric illness influenced the perceived effectiveness of quetiapine. Psychiatric illness was defined as the presence of anxiety, depression, other psychiatric diagnoses, or restless leg syndrome. Multivariable regression techniques were avoided due to insufficient power and sparse data in multiple outcome categories.

Among the 11 participants who provided evaluable responses, a higher proportion of females (5/5, 100%) reported quetiapine to be effective compared to males (2/6, 33.3%). This difference approached but did not reach statistical significance (Fisher’s Exact Test, *p* = 0.061). When stratified by psychiatric illness, participants with a psychiatric diagnosis had a similar rate of reporting effectiveness (4/7, 57.1%) compared to those without (3/4, 75%), with no statistically significant difference observed (Fisher’s Exact Test, *p* = 1.0). The most frequent side effects were sleepiness and weight increase, but because there were only 14 users, our regression models lacked sufficient power to allow for reliable multivariable correction. One significant drawback that should be taken into account when evaluating the results is the limited statistical power.

## Discussion

In this study, we focused on exploring the prevalence of insomnia, poor sleep quality, and the use of sleep aids among medical students, aiming to understand how these sleep-related disorders intersect with chronic conditions and the overall students’ well-being. Our findings highlight a critical and often overlooked aspect of medical education: the pervasive disruption of sleep health, which may significantly hinder academic performance, clinical efficiency, and long-term professional development.

Insomnia symptoms were reported by 80% of participants, while 93.7% were categorized as having “poor” sleep quality according to the PSQI scoring system. This observation reflects the multidimensional nature of the PSQI, which evaluates not only the three domains of insomnia, but also sleep latency, sleep efficiency, nighttime disturbances, and daytime dysfunction [15]. Furthermore, data from the current study showed no significant correlation between the presence of chronic illnesses and PSQI scores, suggesting that poor sleep quality is widespread regardless of comorbid physical or mental conditions. These findings align with existing literature indicating that medical students are particularly vulnerable to sleep disturbances due to high academic demands, extended studying hours, irregular routines, and psychological stress [17, 18]. However, it is important to note that while the PSQI is a robust screening instrument for poor sleep quality, a high score is not equivalent to a clinical diagnosis of a sleep disorder, which would require a comprehensive clinical evaluation. Notably, despite the high prevalence of insomnia symptoms and poor sleep quality in our sample, only 8.8% of participants reported seeking medical advice for their sleep problems. This striking discrepancy highlights a significant treatment gap that warrants attention. Although our questionnaire did not include specific items probing the reasons behind this low help-seeking rate, several barriers likely contribute to the low rate of help-seeking behavior observed in our study (only 8.8%). First, the normalization of poor sleep and fatigue within medical training culture may lead students to view insomnia as a temporary or unworthy concern. Second, time constraints, high workload, and a lack of flexible appointment hours may prevent students from accessing mental health or sleep services. Third, stigma remains a major barrier—medical students may fear judgment or fear that disclosing mental health or sleep problems could harm their academic progress or professional image [19]. In many institutes, there are no dedicated student-only clinics that foster a stigma-free environment for students to seek help. Instead, students in need of support are typically referred to the university’s general psychiatric clinics, which often fail to guarantee privacy or address the specific concerns of students. At the institute of the current study, counseling services were arranged, but they lacked consistency. Frequent physician rotation meant that students often consulted different providers at each visit, preventing the establishment of rapport and hindering long-term follow-up. Similarly, although the faculty had established mentorship programs years ago, these were mainly focused on academic advice. Faculty members were not sufficiently trained to identify early signs of psychological distress, sleep disturbances, or burnout. This differs from the peer-led mentorship program at some universities, such as the University of Cincinnati College of Medicine (University of Cincinnati College of Medicine) [20], where first-year students were mentored by second-year students and the University of Toronto’s same-day counseling services (University of Toronto Student Life) [21]. That approach facilitated a smoother transition into medical school, reduced anxiety, and fostered a stronger sense of belonging, making it more relatable and effective for new students. Similarly, Harvard Medical School integrates structured support systems such as the CAMHS Cares Line, which provides immediate access to counseling and guidance (Harvard University Health Services) [22]. Adding to these institutional gaps, students and trainees also face a heavy academic and clinical workload, which leaves them with limited opportunities to seek timely help.

Taken together, these factors highlight significant deficiencies in the current setting. When compared with international models, these shortcomings become even more apparent, emphasizing the urgent need for sustainable, student-centered mental health services within medical education systems.

The final limitation is the limited awareness about non-pharmacological treatment options like CBT-I, which may leave students relying on ineffective or risky self-medication practices. The low help-seeking behavior among medical students may also be caused by institutional and structural constraints in addition to individual-level ones. These include the absence of confidential, flexible-access clinics on campus, the lack of specific student mental health services or their limited availability, and the scant inclusion of mental health promotion in the medical school curriculum. Additionally, there are less options for early intervention and referral because professors and administrative staff are rarely trained to recognize students who are having sleep issues or mental health issues. Addressing these systemic barriers is essential to creating a supportive environment that promotes student well-being and encourages timely help-seeking. Future research should explore these institutional challenges to inform the development of targeted, evidence-based interventions for medical students.

Our regression analysis showed no statistically significant correlation between the presence of insomnia symptoms and global PSQI scores. While this may seem counterintuitive, it is important to clarify that the analysis assessed the relationship between self-reported insomnia symptoms and the continuous PSQI score, rather than the binary classification of poor sleep quality. Several factors may explain this lack of association. First, the mere presence of insomnia symptoms may not reflect the severity of poor sleep as quantified by the PSQI. Second, although insomnia symptoms align with certain PSQI components (e.g., sleep latency and subjective sleep quality), other components—such as sleep medication use and daytime dysfunction—capture broader constructs that may be influenced by unrelated factors. Finally, response bias or a mismatch between subjective insomnia reporting and the structured format of the PSQI may have attenuated the observed relationship. Future research should apply multivariable models and incorporate validated insomnia scales to more accurately capture the complex interplay between subjective symptoms and overall sleep quality.

Cognitive performance is tightly linked to sleep integrity; even subtle deficits in sleep can lead to declines in memory consolidation, attention span, and executive functioning, all of which are essential for medical education and clinical decision-making. Therefore, sleep disruptions can lead to daytime fatigue, impaired concentration, emotional instability, and decreased academic productivity—factors that can severely impair a student’s ability to learn, perform, and provide patient care [3, 4].

Psychiatric conditions—especially anxiety and depression—were highly prevalent in our study and are closely linked with sleep disturbances. A total of 29.2% (480 participants) were diagnosed with at least one psychiatric illness particularly: anxiety, depression, OCD, ADHD, obstructive sleep apnea, or other non-specified psychiatric conditions. These conditions frequently present with insomnia or non-restorative sleep as core symptoms. The high burden of untreated or sub-optimally managed psychiatric symptoms may perpetuate poor sleep, creating a vicious cycle that exacerbates both psychological and academic stress [23, 24].

The widespread use of psychiatric and neurological medications may underestimate the actual need or use for sleep aids, as only 158 participants explicitly reported using sleep aid medications, while a much larger cohort was using drugs with known sedative side effects. However, these medications appeared insufficient in supporting sleep, as evidenced by the high prevalence of poor sleep quality across the cohort. This suggests that many students may be experiencing unresolved sleep disturbances that are incidentally exposed to sedating agents rather than receiving targeted treatment for insomnia.

Quetiapine was used off-label by 14/17 students as a sleep aid. However, its efficacy remains questionable in this context. The relatively low number of participants reporting quetiapine use for insomnia—and even fewer reporting satisfactory outcomes—limits our ability to draw strong conclusions. One significant drawback that should be taken into account when evaluating the results is the limited statistical power.

More importantly, this off-label use raises concerns regarding long-term safety, as quetiapine is associated with metabolic side effects, cognitive impairment, and dependency risks, particularly when used without psychiatric indication [9]. Confounding by indication is also likely to occur. Most quetiapine utilizers had underlying psychiatric disorders, making it difficult to determine whether adverse effects stemmed from quetiapine or the underlying condition itself. Furthermore, concurrent use of additional sedatives also raises the possibility of polypharmacy confounding, which our research was unable to completely separate, such as, SSRIs, benzodiazepines, antihistamines, which introduces the possibility of drug- drug interactions as well. Given these methodological constraints, our pharmacovigilance results should be viewed as a signal rather than causal evidence. Larger, prospective pharmacoepidemiologic studies that appropriately account for psychiatric comorbidity and polypharmacy are needed to clarify these associations. Yet, though our results cannot be used to draw conclusions regarding the safety or effectiveness of quetiapine in this demographic, the off-label use of the medication for sleep in medical students raises concerns about this practice and calls for more careful monitoring and focused educational initiatives.

Chronic illnesses commonly associated with sleep disruption, such as anxiety, depression, gastroesophageal reflux disease (GERD), restless leg syndrome (RLS), and epilepsy, were also present among our participants. GERD, for instance, is known to interfere with sleep due to nocturnal reflux symptoms [25]. RLS, which may be exacerbated by the prolonged sedentary behavior typical of medical students during long lectures and study sessions, could result in discomfort and insomnia, as well [26, 27]. Epilepsy, while less common, introduces additional complexity, as medication side effects and the unpredictability of seizures can impair both sleep and academic function [28].

Another significant concern is the underuse of non-pharmacological sleep interventions. While pharmacological solutions are frequently relied upon, they often provide only short-term relief. Cognitive Behavioral Therapy for Insomnia (CBT-I), mindfulness, stress management programs, and structured sleep hygiene education have shown substantial evidence in improving sleep outcomes and mental resilience in students [19]. Integrating these strategies into student support services could offer safer and more sustainable long-term solutions.

Although the PSQI analysis revealed no significant sex-based differences in overall sleep quality, females in the study reported significantly higher rates of psychiatric disorders, particularly anxiety, which are known contributors to sleep dysfunction. This may reflect a greater vulnerability of female students to psychological stressors, possibly influenced by hormonal fluctuations, menstrual-related symptoms, and sociocultural pressures [29–31]. At the same time, the lower reported prevalence of psychiatric disorders among males may point to an underestimation driven by stigma or reluctance to seek psychological help. These findings highlight the need for tailored mental health interventions that address sex-specific risk factors and barriers to care.

Building on these findings, peer-led mentorship program, where upper-year medical students guide first- and second-year students academically while conducting informal wellbeing check-ins, could help normalize conversations around stress, sleep, and life balance. The integration of Sleep and Wellbeing courses into the curriculum—delivered as short modules embedded within existing lectures—would enhance students’ literacy on sleep hygiene, stress management, and mental health awareness. Complementing this, quarterly two-hour awareness sessions for both teaching staff and students could improve recognition of sleep hygiene principles and early warning signs of mental distress. To ensure early detection, each department could assign one trained faculty member to act as a wellbeing counselor, offering confidential support to at-risk students. In addition, establishing weekly drop-in wellness hours, where students can meet a counselor, peer supporter, or faculty member without prior booking, would increase service accessibility and visibility.

Leveraging technology, a digital platform with anonymous booking and access to evidence-based self-help tools (e.g., CBT-I modules) would provide a private and scalable method for students to seek help. Finally, the effectiveness of these interventions could be evaluated through pilot programs using validated measures, such as the PSQI, comparing pre- and post-intervention scores [32].

However, one limitation of this study is relying on web-based communication channels to reach target population. While these platforms provided extensive reach, yet potential biases including the overrepresentation of students who are active on social media and underrepresentation of those less engaged with digital communication tools may have arisen.

## Conclusion

This study reveals a high prevalence of insomnia symptoms and poor sleep quality among medical students, with 93.7% classified as poor sleepers. Despite low reported use of sleep aids, many students were taking sedative medications for other conditions, suggesting unmet sleep needs. The off-label use of quetiapine among the small subset of our sample, was limited and largely ineffective, raising concerns about safety and inappropriate prescribing. Higher rates of psychiatric disorders in female students highlight the need for tailored mental health support. Overall, comprehensive, non-pharmacological strategies, such as: structured education on sleep hygiene, access to CBT-I, flexible on-campus counseling services, peer support networks, and campaigns to destigmatize help-seeking behavior, are essential to improve sleep, well-being, and academic performance in this population. To bridge the identified care gap, we propose a multi-tiered student health support program integrated within the medical school structure. In addition, a multi-level intervention plan should be used to close the large care gap and promote the wellbeing of students. One such initiative might be a peer-led sleep health pilot program wherein experienced upper-year students provide organized assistance, psychoeducation, and referral information. Workshops for faculty development might be implemented to assist teachers in identifying and reacting to students' sleep or mental health issues. Lastly, the student portal's incorporation of a digital self-screening tool would enable automated referrals to support services and a private evaluation of sleep issues.

## Data collaborators

We would like to acknowledge the valuable contribution of the following individuals for their efforts in data collection, which significantly supported the progress of this study: Momaen Adel Fathy, Marwa Ismail Selim Hussin, Ahmed Amin Shaaban, Amr Yaser, Mohammed Ismail Maarouf, Arwa Latif, Jana Amr Allam, Mohammed Adel AbdelAlim Awad, Nahla Genidy, Khaled Amir Ibrahim Hamza, Mariam Satour, Toka Osama Abdelaziz, Nourhan ElSayed Mohamed, Mohamed T. G. Hassan, Sama Osama Abdellatif Khatib, and Natalie Nashed.

## Supplementary Information

Supplementary Material 1.

## Data Availability

The datasets used and/or analysed during the current study are available from the corresponding author on reasonable request.
