# Supplementary material for: Too tired to learn: insomnia, sleep quality, and sleep aid practices among Kasr Al Ainy medical students: a cross-sectional analysis
Source: BMC Med Educ. 2025 Nov 6;25:1553. doi: 10.1186/s12909-025-08054-1 (PMC12590862; doi:10.1186/s12909-025-08054-1)
Supplement: Supplementary file 1 — Supplementary Material 1. [file 12909_2025_8054_MOESM1_ESM.pdf]

This form is directed to Medical students and Physicians at Faculty Of Medicine Cairo University

For medical students, physicians, and interns at faculty of medicine cairo university , please answer the following questions as a part of a conducted survey about insomnia. By completing the survey, you agree to share your answers (anonymously) to be used in this ongoing study.

* تشير إلى أنّ السؤال مطلوب

1. Collaborator ID number

____

General information

2. 1. Are you a male or female?*

حدد دائرة واحدة فقط.

- Male
- Female

3. 2. How old are you?*

____

4. 3. Are you a medical student, physician, or intern?

حدد دائرة واحدة فقط.

- Medical student
- Physician
- Intern

5. 4. What is your current studying grade/or specialty ?*

____

6. 5. Do you suffer from any of these diseases?*

حدد كل الإجابات الملائمة.

- Diabetes
- Hypertension
- Asthma
- Cancer
- Anemia
- Thyroid disorders
- Autoimmune diseases
- Sinusitis
- Eye disorders
- Anxiety
- Depression
- Other psychiatric illness
- Restless leg syndrom
- Obstructive sleep apnea
- Gastroesophageal reflux disease
- Neurological disorder
- None
- أخرى: ______________________________

7. 6. If you suffer from any of the previous diseases, do you receive any regular medications for this condition(s)? *

حدد دائرة واحدة فقط.

- Yes
- No
- Not applicable (I don't suffer from any chronic disease)
- أخرى: ______________________

8. 7. If you answered (Yes) for the previous question, please specify the medications are receiving.

____

____

____

____

____

9. 8. Do you suffer from any of these symptoms of insomnia? check all applicable answers *

حدد كل الإجابات الملائمة.

- [ ] Find it hard to go to sleep
- [ ] Lie awake at night
- [ ] Waking up too early
- [ ] Still feel tired after waking up
- [ ] Feel tired and irritable during the day
- [ ] Find it hard to nap during the day even though you're tired
- [ ] It is hard for you to concentrate
- [ ] Having ongoing worries about sleep.
- [ ] None

10. 9. If you answered yes for any of the previous symptoms in question "8", have you sought medical advice for your condition (insomnia)? *

حدد دائرة واحدة فقط.

- Yes
- No
- Not applicable (I don't suffer from any of these symptoms)

11. 10. If you suffer from insomnia, have you been prescribed any sleep aids before?*

حدد دائرة واحدة فقط.

- Yes I have been prescribed sleep aids*التخطي إلى السؤال 12*
- I take a sleep aid, but without a medical prescription*التخطي إلى السؤال 12*
- I didn't seek for treatment despite being suffering from insomnia *التخطي إلى السؤال 18*
- No, I wasn't prescribed a sleep aid, rather I was advised with behavioral therapy or educated about proper sleep habits*التخطي إلى السؤال 18*
- Not applicable (I don't suffer from any of insomnia symptoms) *التخطي إلى السؤال 18*
- Option 6

التخطي إلى السؤال 12

Sleep aids

12. 11. If you are currently receiving or received a sleep aid before, how long have you been receiving/received these medications? *

حدد كل الإجابات الملائمة.

- [ ] Days
- [ ] Weeks
- [ ] Months
- [ ] On demand
- [ ] Not applicable

13. 12. Have you tried any of these sleep aids? check all applicable answers*

حدد كل الإجابات الملائمة.

- [ ] Melatonin
- [ ] Quetiapine (serquel)
- [ ] zaleplon (Sonata)
- [ ] zolpidem (Zolpimist)
- [ ] doxepin (Silenor)
- [ ] secobarbital (Seconal)
- [ ] ramelteon (Rozerem)
- [ ] temazepam (Restoril)
- [ ] daridorexant (Quviviq)
- [ ] eszopiclone (Lunesta)
- [ ] zolpidem (Intermezzo)
- [ ] tasimelteon (Hetlioz)
- [ ] triazolam (Halcion)
- [ ] Bromazepam (calmepam)
- [ ] Flurazepam
- [ ] Lorazepam (xanax)
- [ ] Estazolam
- [ ] quazepam (Doral)
- [ ] lemborexant (Dayvigo)
- [ ] butabarbital (Butisol)
- [ ] suvorexant (Belsomra)
- [ ] diphenhydramine (Benadryl)
- [ ] herbals
- [ ] None
- [ ] أخرى: ____________________

14. 13. Have you received quetiapine (Seroquel) as a sleep aid before?*

حدد دائرة واحدة فقط.

- Yes*التخطي إلى السؤال 15*
- No*التخطي إلى السؤال 18*
- I received quetiapine, but for other indication*التخطي إلى السؤال 15*

التخطي إلى السؤال 15

Quetiapine

15. 14. What is the dose of quetiapine you have been receiving/ received before?

حدد دائرة واحدة فقط.

- 25 mg at bed time
- 50 mg at bed time
- 100 mg at bed time
- Not applicable (I didn't receive quetiapine before)
- أخرى: ______________________

16. 15. If you are currently receiving or have received **Quetiapine** as a sleep aid before, have you suffered from any of the following symptoms? (Check all applicable answers)

حدد كل الإجابات الملائمة.

- [ ] Failure to control insomnia
- [ ] Drowsiness
- [ ] Sedation
- [ ] Lack of concentration
- [ ] metabolic changes
- [ ] Increased appetite
- [ ] Weight gain
- [ ] Extrapyramidal side effects
- [ ] Menstrual changes
- [ ] Palpitations
- [ ] None
- [ ] أخرى: ______________________

17. 16. Did quetiapine effectively managed your insomnia

حدد دائرة واحدة فقط.

- ( ) Yes, I strongly agree
- ( ) Yes, I agree
- ( ) I'm not Sure
- ( ) No

This section will qualify the sleep quality using the Pittsburgh Sleep Quality Index (PSQI)

18. 1.During the past month, what time have you usually gone to bed at night?

____

19. 2.During the past month, how long (in minutes) has it usually taken you to fall asleep each night? _

20. 3. During the past month, what time have you usually gotten up in the morning?

21. 4. During the past month, how many hours of actual sleep did you get at night? (This may be different than the number of hours you spent in bed.)

22. 6. During the past month, how often have you had trouble sleeping because can't get to sleep within 30 minutes

حدد دائرة واحدة فقط.

- Not during the past month
- Less than once a week
- Once or twice a week
- Three or more times a week

23. 7. Wake up at the middle of the night or early morning

حدد دائرة واحدة فقط.

- Not during the past month
- Less than once a week
- Once or twice a week
- Three or more times a week

24. 8. Have to get up to use the toilet

حدد دائرة واحدة فقط.

- Not during the past month
- Less than once a week
- Once or twice a week
- Three or more times a week

25. 9. Can't breathe comfortably

حدد دائرة واحدة فقط.

- Not during the past month
- Less than once a week
- Once or twice a week
- Three or more times a week

26. 10. Cough or snore loudly

حدد دائرة واحدة فقط.

- Not during the past month
- Less than once a week
- Once or twice a week
- Three or more times a week

27. 11. Feel too cold

حدد دائرة واحدة فقط.

- Not during the past month
- Less than once a week
- once or twice a week
- Three or more times a week

28. 12. Feel too hot

حدد دائرة واحدة فقط.

- Not Once
- Less than once a week
- Once or twice a week
- Three or more times a week

29. 13. Have bad dreams

حدد دائرة واحدة فقط.

- Not during the past month
- Less than once a week
- Once or twice a week
- Three or more times a week

30. 14. Have pain

حدد دائرة واحدة فقط.

- Not during the past month
- Less than once a week
- Once or twice a week
- Three or more times a week

31. 15. Can't sleep for other reason, describe in the others

حدد دائرة واحدة فقط.

- Not during the past month
- Less than once a week
- Once or twice a week
- Three or more times a week
- أخرى: ______________________

32. 16. During the past month, how often have you taken medicine to help you sleep (prescribed or "over the counter")?

حدد دائرة واحدة فقط.

- Not during the past month
- Less than once a week
- Once or twice a week
- Three or more times a week

33. 17. During the past month, how often have you had trouble staying awake while driving, eating meals, or engaging in social activity?

حدد دائرة واحدة فقط.

- Not during the past month
- Less than once a week
- Once or twice a week
- Three or more times a week

34. 18. During the past month, how much of a problem has it been for you to keep up enough enthusiasm to get things done?

حدد دائرة واحدة فقط.

- Not a problem at all
- Only a very slight problem
- Somewhat of a problem
- A very big problem

35. 19. During the past month, how would you rate your sleep quality overall?

حدد دائرة واحدة فقط.

- Very good
- Fairly good
- Fairly bad
- Very bad

36. 20. Do you have a bed partner or room mate?

حدد دائرة واحدة فقط.

- No bed partner or room mate
- Partner/room mate in other room
- Partner in same room, but not same bed
- Partner in same bed

37. 21. If you have a bed partner or room mate, ask him/her how often in the past month you have had: loud snoring

حدد دائرة واحدة فقط.

- Not during the past month
- Less than once a week
- Once or twice a week
- Three or more times a week

38. 22. If you have a bed partner or room mate, ask him/her how often in the past month you have had: long pauses between breaths while asleep

حدد دائرة واحدة فقط.

- Not during the past month
- Less than once a week
- Once or twice a week
- Three or more times a week

39. 23. If you have a bed partner or room mate, ask him/her how often in the past month you have had: legs twiching or jerking while you sleep

حدد دائرة واحدة فقط.

- Not during the past month
- Less than once a week
- Once or twice a week
- Three or more times a week

40. 24. If you have a bed partner or room mate, ask him/her how often in the past month you have had: Episodes of disorientation or confusion during sleep

حدد دائرة واحدة فقط.

- Not during the past month
- Less than once a week
- Once or twice a week
- Three or more times a week

41. 25. If you have a bed partner or room mate, ask him/her how often in the past month you have had: other restlessness while you sleep, please describe in the other option

حدد دائرة واحدة فقط.

- Not during the past month
- Less than once a week
- Once or twice a week
- Three or more times a week
- أخرى: ____________________

Others

42. 1. Do you feel sleepy during the daytime when you are not busy?

حدد دائرة واحدة فقط.

- Never
- Sometimes
- Often

43. 2. Do you worry about falling asleep, staying asleep or getting enough sleep?

حدد دائرة واحدة فقط.

- Yes
- No
- Sometimes

44. 3. Do you fall asleep in the daytime against your will?

حدد دائرة واحدة فقط.

- Never
- Sometimes
- Often
- Yes, daily

45. 4. Do you consume (food, drinks, medications) before bedtime? (If yes, specify their nature/type and amount)

____

46. 5. Do you have regular limb or body movements in bed at night? If yes, state how frequency

47. 6. To what extent do you consider your sleep problem to interfere with your daily functioning (e.g., daytime fatigue, ability to function at work, daily chores, concentration, memory, mood, etc.)?

حدد دائرة واحدة فقط.

- Not At all Interfering
- A little
- Much

48. 7. Do you think your sleeping problem is impairing your quality of life?

حدد دائرة واحدة فقط.

- Not at all
- Little
- Much

49. 8. How worried/distressed are you about your current problem?

حدد دائرة واحدة فقط.

- Not at all
- Little
- Much

50. 9. Do you have any new or ongoing stresses related to work, personal problems, or any other issue?

حدد دائرة واحدة فقط.

- Yes
- No
- Not sure

51. 10. Do you work night shifts?

حدد دائرة واحدة فقط.

- Yes, frequently
- Yes, occasionally
- No
- Not applicable

52. 11. Do you consume alcohol, tobacco, or caffeine

حدد كل الإجابات الملائمة.

- [ ] Yes, alcohol
- [ ] Yes, tobacco
- [ ] Yes, coffee, espresso, ..etc.
- [ ] Yes, black/green tea, tea with milk
- [ ] Yes, energy drinks
- [ ] Yes, sodas
- [ ] Yes, cocoa chocolate
- [ ] No

53. 12. Do you exercise regularly?

حدد دائرة واحدة فقط.

Yes

No

لم يتم إنشاء هذا المحتوى ولا اعتماده من قِبل Google.

نماذج Google
